# Early antibiotics administration during targeted temperature management after out-of-hospital cardiac arrest: a nationwide database study

**DOI:** 10.1186/s12871-016-0257-3

**Published:** 2016-10-07

**Authors:** Takashi Tagami, Hiroki Matsui, Masamune Kuno, Yuuta Moroe, Junya Kaneko, Kyoko Unemoto, Kiyohide Fushimi, Hideo Yasunaga

**Affiliations:** 1 Department of Clinical Epidemiology and Health Economics, School of Public Health, Graduate School of Medicine, The University of Tokyo, 7-3-1 Hongo, Bunkyo-ku, Tokyo, 1138555 Japan; 2 Department of Emergency and Critical Care Medicine, Nippon Medical School Tama Nagayama Hospital, 1-7-1 Nagayama, Tama-shi, Tokyo 2068512 Japan; 3 Department of Health Informatics and Policy, Graduate School of Medicine, Tokyo Medical and Dental University, 1-5-45 Yushima, Bunkyoku, Tokyo, 1138510 Japan

**Keywords:** Antibiotics, Cardiac arrest, Extracorporeal membrane oxygenation, Infection, Targeted temperature management

## Abstract

**Background:**

Patients resuscitated after cardiac arrest are reportedly at high risk for infection and sepsis, especially those treated with targeted temperature management (TTM). There is, however, limited evidence suggesting that early antibiotic use improves patient outcomes. We examined the hypothesis that early treatment with antibiotics reduces mortality in patients with cardiac arrest receiving TTM.

**Methods:**

We identified 2803 patients with cardiogenic out-of-hospital cardiac arrest (OHCA) that were treated with TTM and were admitted to 371 hospitals that contribute to the Japanese Diagnosis Procedure Combination inpatient database between July 2007 and March 2013. Of these, 1272 received antibiotics within the first 2 days (antibiotics) and 1531 did not (control). We generated 802 propensity score-matched pairs.

**Results:**

There was no significant difference in 30-day mortality between the groups (control vs. antibiotics; 33.0 % vs. 29.9 %; difference, 3.1 %; 95 % confidence interval [CI], −1.4 to 7.7 %, *p* = 0.18). Analysis using the hospital antibiotics prescribing rate as an instrumental variable showed that antibiotic use was not significantly associated with a reduction in 30-day mortality (6.6 %, CI 95 %, −0.5 to 13.7 %, *p* = 0.28). A subgroup analysis of patients who required extracorporeal membrane oxygenation (ECMO) indicated a significant difference in 30-day mortality between the 2 groups (62.9 % vs. 43.5 %; difference 19.3 %, CI 95 %, 5.9 to 32.7 %, *p* = 0.005). In the instrumental variable model, the estimated reduction in 30-day mortality associated with antibiotics was 18.2 % (CI 95 %, 21.3 to 34.4 %, *p* = 0.03) in ECMO patients.

**Conclusions:**

Although there was no significant association between the use of antibiotics and mortality after overall cardiogenic OHCA treated with TTM, antibiotics may be beneficial in patients who require ECMO.

## Background

Patients with return of spontaneous circulation (ROSC) after out-of-hospital cardiac arrest (OHCA) suffer from prolonged, complete, whole-body ischemia and reperfusion, and have a grim prognosis [1, 2]. Post-cardiac arrest ischemia-reperfusion injury to the brain may reportedly be attenuated by induced hypothermia [3–5]. Thus, the 2015 international guidelines for cardiac arrest recommend a targeted temperature-management (TTM) strategy for adults sustaining an OHCA with an initial shockable rhythm who remain unresponsive after the ROSC [6, 7].

Patients resuscitated after cardiac arrest are reportedly at high risk of developing infectious diseases, especially pneumonia, several days after ROSC [2, 8–13]. Although recent guidelines recommend a TTM strategy [6, 7], hypothermia can impair the immune system and increase the incidence of infection [14, 15]. In addition, invasive medical techniques, such as extracorporeal membrane oxygenation (ECMO), intra-aortic balloon pumping (IABP) and continuous renal replacement therapy (CRRT), are used during TTM and organ support after OHCA [16]. These invasive devices (especially ECMO) may themselves increase the risk of infection [17–19]. Consequently, patients undergoing TTM after cardiac arrest are likely to be at greater risk of infection and death.

Although antibiotic administration is the mainstream treatment for infectious diseases, only a few retrospective studies have suggested a beneficial influence on the survival of OHCA patients who received early antibiotic administration while undergoing TTM [20, 21]. Moreover, there are no current reports of a relationship between early antibiotic administration and mortality among patients with OHCA requiring invasive life-supporting interventions.

We hypothesized that prophylactic systemic antibiotic therapy during TTM may reduce mortality in OHCA patients, especially in those requiring invasive life-supporting interventions. The purpose of this study was to evaluate our hypothesis using a large nationwide inpatient database in Japan.

## Methods

Conduct of the study was approved by the Institutional Review Board of The University of Tokyo. Requirement for informed patient consent was waived because of the anonymous nature of the data.

### Data source and variables

We retrospectively evaluated the Japanese Diagnosis Procedure Combination (DPC) database, which was described previously [16, 22, 23]. Briefly, the DPC database includes administrative claims and discharge abstract data for all inpatients discharged from more than 1000 participating hospitals, covering all 82 academic hospitals and more than 90 % of all tertiary-care emergency hospitals in Japan [16, 22, 23]. The database includes the following information for each patient recorded using a uniform data-submission form: age; sex; primary diagnosis, comorbidities on admission, and post-admission complications coded using the International Classification of Diseases, 10th Revision (ICD-10) codes; medical procedures; daily records of all drugs administered and devices used; and discharge status. Patient follow-up began on the day of admission and ended on the date of discharge, either to home, to another hospital, or because of death. Patients with cardiac arrest upon arrival at the hospital were defined as experiencing OHCA, with chest compressions performed on/after arrival. We assumed that cardiac arrest was of cardiac aetiology unless there was an obvious non-cardiac cause (i.e. cerebrovascular disease, respiratory disease, severe trauma, drowning, asphyxiation or drug overdose) [16], in accordance with the International Liaison Committee on Resuscitation consensus statement [24, 25]. We defined patients with non-cardiogenic cardiopulmonary arrest as follows: ICD-10 codes at primary diagnosis and comorbidities upon admission of J60 to J61.9 [subarachnoid and cerebral haemorrhage], I71.0 to I71.9 [aorta dissection and aneurysm], I26.0 to I26.9 [pulmonary embolism], J45.9 and J46 [severe asthma], S00 to T98 [trauma, burns, hanging, accidental hypothermia, drowning, electrocution, anaphylaxis, drug overdose], J69 and T71 [asphyxiation], or K25.0, K25.2, K26.0, K26.2, K27.0, K27.2, K28.0, K28.2, K92.0 to 92.2, and I85.0 to I85.9 [acute gastrointestinal bleeding or oesophageal/gastric varices]).

We designated hospital volume as the number of eligible patients treated for the current study and categorized hospitals into tertiles (i.e. low, medium, and high). The hospital type was categorized as academic or non-academic. The Japanese Ministry of Health, Labour and Welfare officially approved TTM for patients with cardiac arrest only (i.e. not in other disorders such as traumatic brain injury) in April 2006.

### Patient selection and end point

Adults with cardiogenic OHCA were identified in the DPC database from July 2007 to March 2013. We did not include in-hospital cardiac arrest cases in this study. The inclusion criteria were as follows: (i) age ≥18 years; (ii) diagnosis of OHCA (ICD-10 codes at primary diagnosis and comorbidities on admission of I46.0 [cardiac arrest with successful resuscitation], I46.1 [sudden cardiac death], or I46.9 [cardiac arrest, unspecified]); (iii) and administration of therapeutic hypothermia on days zero and one. Exclusion criteria were as follows: (i) patients with non-cardiogenic cardiopulmonary arrest; (ii) patients with malignancy, caducity, and pneumonia upon admission (to maintain comparability between the groups; C00 to C97 [malignancy], R54 [caducity], J13 to J18, and J690 [bacterial pneumonia and aspiration pneumonia]); (iii) patients discharged within 2 days of admission (to avoid immortal time bias [26]). Thus, we compared patients who received antibiotics within 2 days of admission (antibiotics group) and those who did not receive any antibiotics within that time (control group) [23].

The main endpoint used in this study was all-cause, 30-day in-hospital mortality [25]. We performed subgroup analyses comparing mortality between other groups. We also evaluated the incidence of pneumonia emergence after admission between the two groups.

### Statistical analyses

We performed propensity scoring and instrumental variable analyses. To estimate the propensity score, we fit a logistic regression model designed for antibiotic treatment as a function of background-patient characteristics and in-hospital treatments or interventions performed on day 0. We included the following: age; sex; hospital type; hospital volume; fiscal year; cardiac arrest on hospital arrival; administration of epinephrine; ventricular fibrillation; defibrillation performed; requirement for PCI, ECMO, IABP and/or CRRT; requirement for catecholamines or vasopressin; and requirement for antiarrhythmic drugs, sivelestat sodium use, and blood transfusion. We performed a one-to-one matched analysis using nearest-neighbour matching based on the estimated propensity scores of the patients. A match occurred when a patient in the antibiotics group had an estimated score within 0.2 standard deviations of a patient in the control group [27]. We examined the balance in baseline variables using standardized differences, where >10 % was regarded as imbalanced [27]. Descriptive statistics are presented for all patients and propensity score-matched groups. Continuous variables were compared using a t*-*test. Categorical variables were compared using the chi-square test or Fisher’s exact test. We performed logistic regression analysis fitted with generalized estimating equations to examine the association between antibiotics use and survival and accounted for the paired nature of the propensity score-matched patients. We used Cox regression analysis to assess differences in in-hospital survival rates between patients with and without antibiotic treatment in the propensity score-matched groups. Odds ratios (ORs) and hazard ratios (HRs) with 95 % confidence intervals (CIs) were calculated.

We also performed instrumental variable analysis as confirmatory analysis for the propensity score analyses [28]. We used the hospitals’ antibiotics prescribing preference as an instrumental variable, and computed the differences in the risk of 30-day mortality between the groups with and those without early antibiotic administration. This approach was implemented using a 2-stage least-squares method that also adjusted for patient demographic characteristics. We classified hospitals that administered early antibiotics to the 50th percentile or more of eligible patients as hospitals with a preference for early antibiotics administration, and those that administered to less than the 50th percentile of eligible patients as hospitals without a preference for early antibiotics administration. We estimated the risk difference with its 95 % CI. To confirm that the percentage of hospital use of antibiotics was not a weak instrument, we used a partial F test. The null hypothesis was that there was no association between patterns of hospital antibiotics use and actual antibiotics use. An F statistic >10 suggests that the instrument is not weak [28].

We also performed subgroup analyses to identify the patients who may have benefited the most from antibiotics therapy. We selected relevant subgroups among the propensity score-matched patients and estimated their risk ratios and 95 % CIs for 30-day mortality. For robustness, we further evaluated the subgroup of patients who had the most benefit from early antibiotics administration (i.e. the lowest risk ratio group of patients for 30-day mortality), and performed the same analyses as all patients, including Cox regression and instrumental variable analyses.

A *P* value <0.05 was considered statistically significant. All statistical analyses were performed using IBM SPSS version 22 (IBM Corp., Armonk, NY, USA) and Stata/SE 13.0 (Stata Corp., College Station, TX, USA).

## Results

**Fig. 1:**
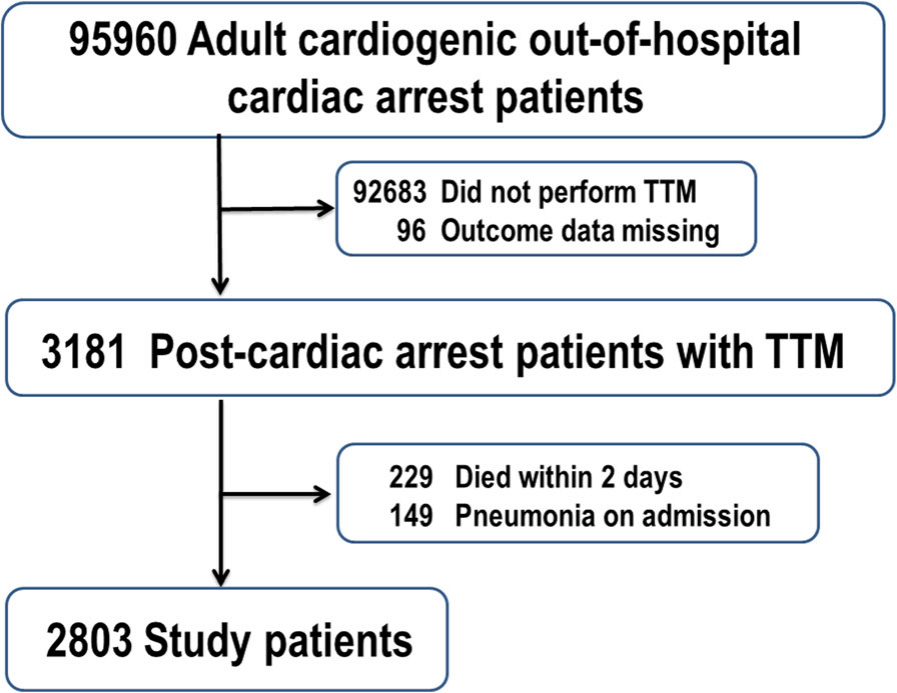
Patient selection

**Fig. 2:**
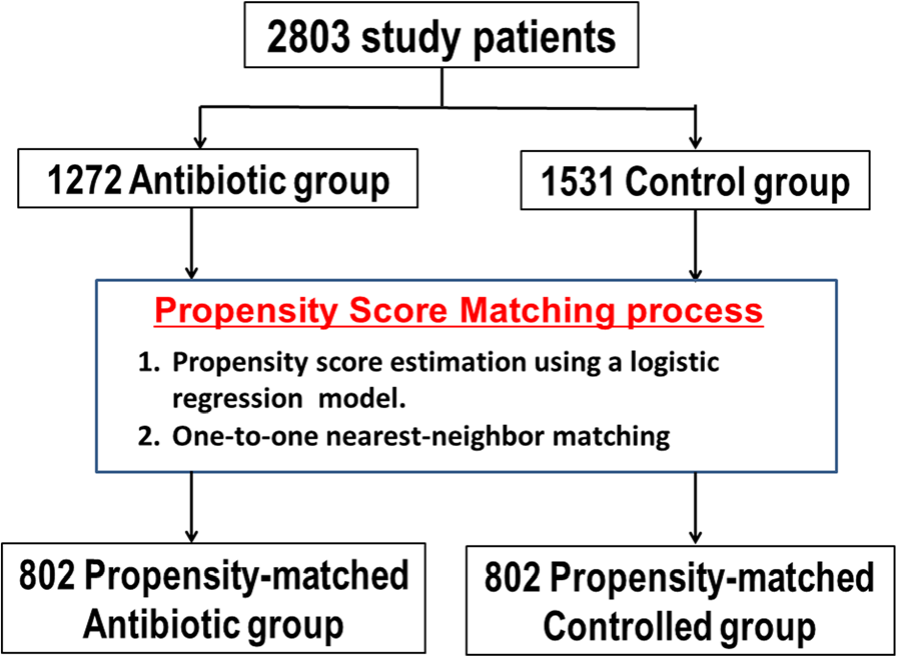
Propensity score matching process

During the study period, 95,960 OHCA patients were identified in the database. Of the patients initially identified, 2803 eligible cardiogenic patients with OHCA were treated with TTM in 371 hospitals (Fig. 1). These patients were categorized into the antibiotic-treated (*n* = 1272) and control groups (*n* = 1531), and 802 propensity score-matched pairs were generated (Fig. 2). Table 1 shows the demographic and clinical characteristics of the patients in the two groups. In the antibiotics group (*n* = 1272), the most common antibiotics administered were as follows: first-generation cephalosporin for 58.6 % (746 patients), third-generation cephalosporin for 10.4 % (145 patients), and broad-spectrum penicillin for 6.3 % (80 patients).

**Table 1:** Patient characteristics, and initial treatments and interventions

	Unmatched groups					Propensity score-matched groups				
Variable	Control (*n* = 1531)		Antibiotics (*n* = 1272)		Standardized differences, %	Control (*n* = 802)		Antibiotics (*n* = 802)		Standardized differences, %
Age, mean (SD)	60.1	(15.3)	60.7	(14.3)	−3.5	60.7	(14.8)	60.6	(14.6)	1.0
Sex (male)^a^	1,151	(75.2)	984	(77.4)	−5.1	613	(76.4)	609	(75.9)	1.2
Academic hospital	620	(40.5)	392	(30.8)	20.3	262	(32.7)	279	(34.8)	−4.5
Hospital volume, cases										
Low, <10	484	(31.6)	505	(39.7)	−16.9	317	(39.5)	305	(38.0)	3.1
Medium 11–22	470	(30.7)	419	(32.9)	−4.8	271	(33.8)	264	(32.9)	1.9
High, >23	577	(37.7)	348	(27.4)	22.2	214	(26.7)	233	(29.1)	−5.3
Ventricular fibrillation	746	(48.7)	645	(50.7)	−4.0	397	(49.5)	413	(51.5)	−4.0
Required defibrillation on admission	328	(21.4)	409	(32.2)	−24.4	186	(23.2)	204	(25.4)	−5.2
Cardiac arrest on admission	540	(35.3)	586	(46.1)	−22.1	320	(39.9)	319	(39.8)	0.3
Epinephrine provided on admission	597	(39.0)	617	(48.5)	−19.3	342	(42.6)	338	(42.1)	1.0
Percutaneous coronary intervention	872	(57.0)	902	(70.9)	−29.4	507	(63.2)	536	(66.8)	−7.6
Intra-aortic balloon pumping	308	(20.1)	483	(38.0)	−40.1	218	(27.2)	204	(25.4)	4.0
Continuous renal replacement therapy	136	(8.9)	248	(19.5)	−30.8	99	(12.3)	104	(13.0)	−1.9
Extracorporeal membrane oxygenation system Pharmacologic intervention	147	(9.6)	298	(23.4)	−37.9	105	(13.1)	108	(13.5)	−1.1
Dopamine	741	(48.4)	703	(55.3)	−13.8	408	(50.9)	425	(53.0)	−4.2
Dobutamine	286	(18.7)	403	(31.7)	−30.3	189	(23.6)	176	(21.9)	3.9
Norepinephrine	487	(31.8)	584	(45.9)	−29.2	300	(37.4)	300	(37.4)	0.0
Vasopressin	51	(3.3)	53	(4.2)	−4.4	28	(3.5)	26	(3.2)	1.4
Amiodarone	371	(24.2)	463	(36.4)	−26.7	234	(29.2)	244	(30.4)	−2.7
Nifekalant	76	(5.0)	103	(8.1)	−12.7	45	(5.6)	57	(7.1)	−6.1
Lidocaine	370	(24.2)	455	(35.8)	−25.5	233	(29.1)	241	(30.0)	−2.2
Sivelestat sodium	44	(2.9)	84	(6.6)	−17.6	32	(4.0)	33	(4.1)	−0.6
Blood transfusion										
Red blood cells	133	(8.7)	268	(21.1)	−35.3	92	(11.5)	99	(12.3)	−2.7
Fresh frozen plasma	86	(5.6)	199	(15.6)	−33.0	59	(7.4)	66	(8.2)	−3.3
Platelets	21	(1.4)	61	(4.8)	−19.9	16	(2.0)	15	(1.9)	0.9

^a^numbers in parentheses are proportions (%) unless otherwise stated

### Overall analysis

**Fig. 3:**
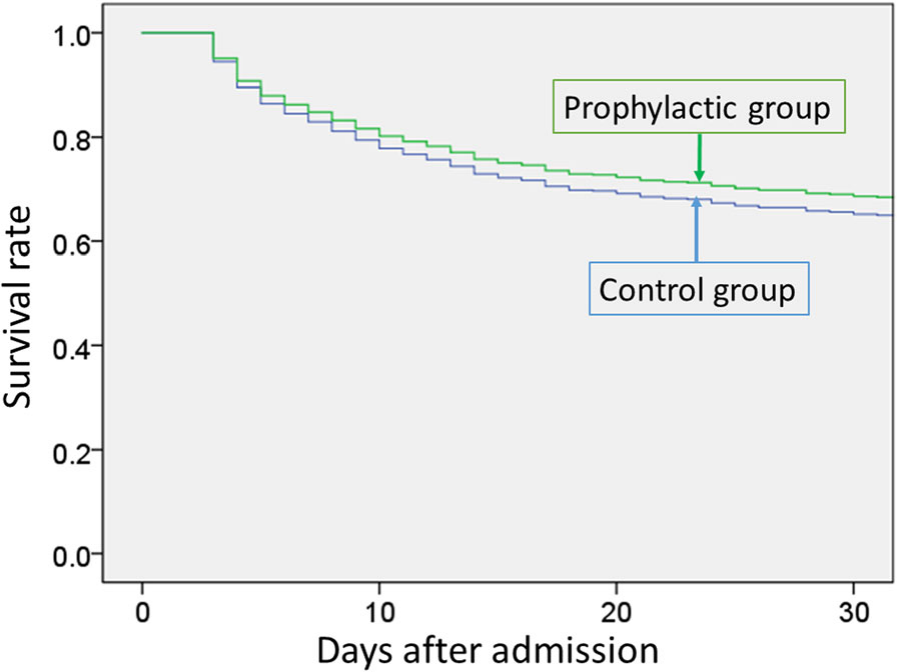
Survival plots for all patients treated with or without early antibiotics administration in propensity score-matched groups. There was no significant difference in 30-day mortality between the antibiotics and control groups. (Cox regression analysis; hazard ratio, 0.88; confidence interval 95 %, 0.75 to 1.04)

The overall 30-day mortality was 32.5 % (910 of 2803 patients), and the incidence of pneumonia after admission was 14.1 % (395 of 2803 patients). Among unmatched patients, significant differences in 30-day mortality were observed between the two groups (30.3 % in the control group compared with 35.1 % in the antibiotics-treated group, difference 4.8 %; CI 95 %, 1.2 to 8.2 %, *p* = 0.007), but not among matched patients (33.0 % versus 29.9 %, respectively, difference 3.1 %; CI 95 %, −1.4 to 7.7 %, *p* = 0.18). Logistic regression analyses showed that there was no significant association between the use of antibiotics and lower 30-day mortality in the propensity score-matched groups (OR 0.89; CI 95 %, 0.73 to 1.1; *p* = 0.24). The Cox regression analysis did not indicate significant differences in-hospital mortality between the control and antibiotics groups for the propensity-matched groups (HR 0.88, CI 95 % 0.75 to 1.04, *p* = 0.12; Fig. 3).

In the instrumental variable model, the null hypothesis of ‘no association between patterns of hospital antibiotics use and actual antibiotics use’ was rejected (*P* < 0.001). Our instrument of pattern of hospital antibiotics use was not weak (F statistic = 702). The estimated reduction in 30-day mortality was not significantly associated with administration of antibiotics (6.6 %, CI 95 %, −0.5 to 13.7 %, *p* = 0.28).

Among unmatched patients, there was a significantly lower incidence of pneumonia after admission in the antibiotic group than in the control group (12.6 % vs. 15.3 %, difference −3.7 %; CI 95 %, −0.2 to −5.3 %, *p* = 0.04). However, this difference was not significant in the matched group (13.0 % vs. 15.8 %, difference −6.3 %; CI 95 %, −0.2 to 0.6 %, *p* = 0.10).

### Subgroup analyses

**Fig. 4:**
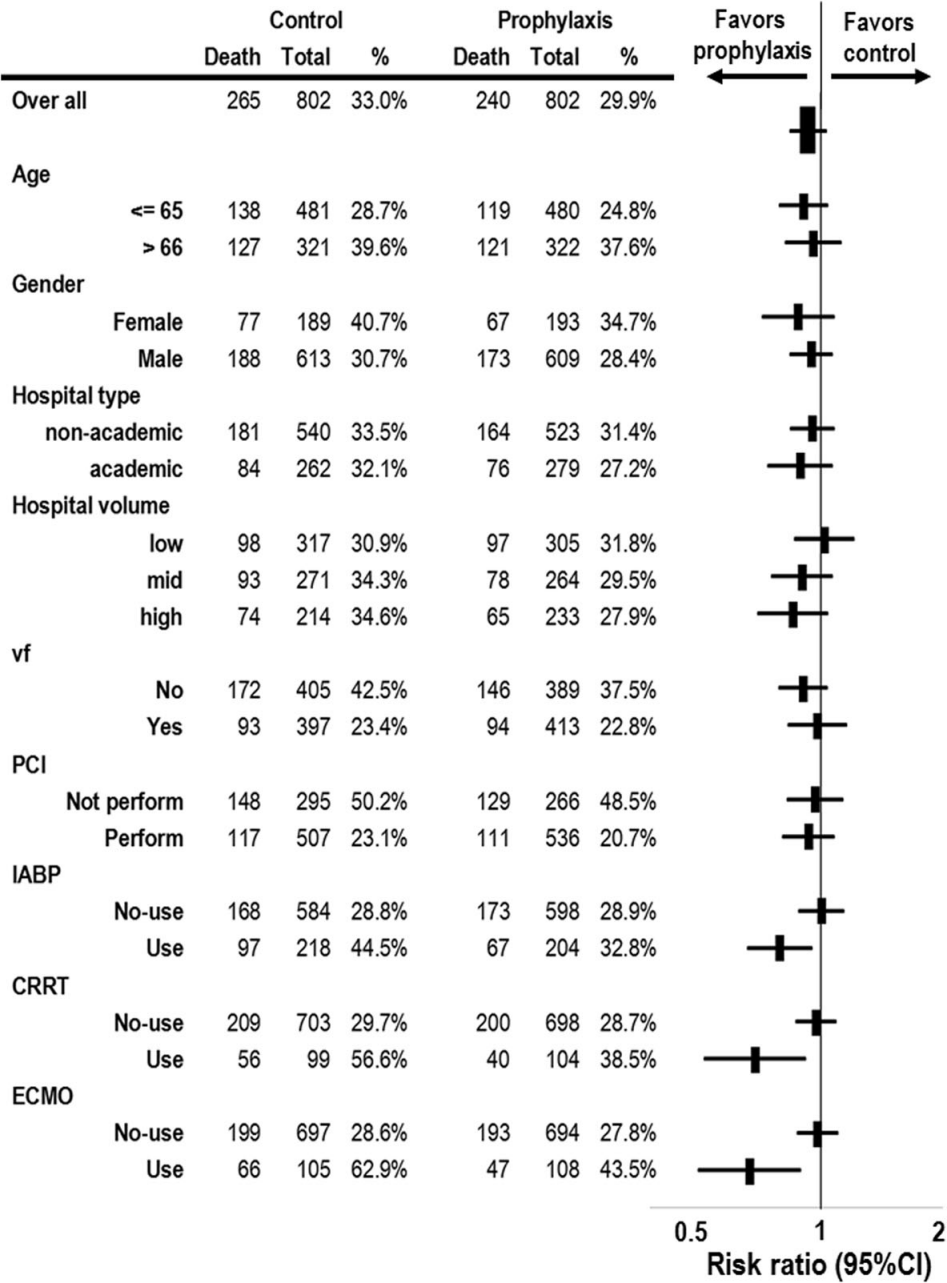
Risk ratios of in-hospital mortality associated with antibiotic use in propensity score-matched patients. CRRT, continuous renal replacement therapy; ECMO, extracorporeal membrane oxygenation; IABP, intra-aortic balloon pumping; PCI, percutaneous coronary angiography/intervention; vf, ventricular fibrillation

The risk of 30-day mortality associated with antibiotics use in the propensity score-matched patients for subgroups is presented in Fig. 4. There was a significant association between the use of antibiotics and lower mortality in the subgroup of patients who required ECMO, IABP, or CRRT. The lowest risk of 30-day mortality associated with the administration of antibiotics was observed in the subgroup who required ECMO (risk ratio 0.67; CI 95 %, 0.50 to 0.89, *p* = 0.006). Among propensity score-matched patients who did not require ECMO, there was no significant difference in 30-day mortality between the two groups (28.6 % in the antibiotics group versus 27.8 % in the control group, difference 0.7 %; CI 95 %, −3.9 to 5.5 %, *p* = 0.76). In contrast, among patients who required ECMO, there was a significant difference in 30-day mortality between the 2 groups (62.9 % in the control group versus 43.5 % in the antibiotics group, difference 19.3 %; CI 95 %, 5.9 to 32.7 %; *p* = 0.005).

**Fig. 5:**
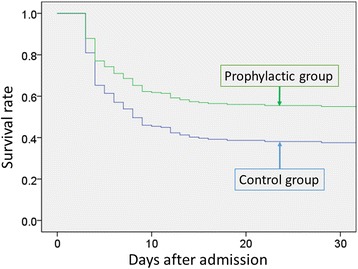
Survival plots for a subgroup of patients who required extracorporeal membrane oxygenation in the propensity score-matched groups. There was a significant difference in 30-day mortality between the antibiotics and control groups (Cox regression analysis; hazard ratio, 0.61; 95 % confidence interval, 0.43 to 0.87)

Among patients who required ECMO, a logistic regression analysis indicated that there was a significant association between the the early use of antibiotics and lower 30-day mortality in the propensity score-matched groups (OR 0.47, CI 95 %, 0.27 to 0.83, *p* = 0.009). Cox regression analysis showed significant mortality differences between the control and antibiotics groups for the propensity-matched groups (HR 0.61, CI 95 %, 0.43 to 0.87, *p* = 0.005; Fig. 5). In the instrumental variable model, the estimated reduction in 30-day mortality associated with receipt of antibiotics was 18.2 % (CI 95 %, 2.1 to 34.4 %, *p* = 0.03). The lower incidence of pneumonia after admission in the early antibiotics-treated group than in the control group was not significant among patients who required ECMO (9.3 % vs. 13.3 %, difference −4.1 %; CI 95 %, −12.6 to 4.4 %, *p* = 0.35).

## Discussion

The results of this study using a nationwide database suggest that there was no significant association between the use of early antibiotics administration and survival in a cohort of patients with OHCA receiving TTM. However, we found a significant association between the use of antibiotics and lower mortality in the subgroup of patients who required ECMO, IABP, or CRRT.

We analysed real-world clinical data of more than 2,800 adults with cardiogenic OHCA treated at 371 hospitals. In the present study, the decision to provide antibiotics was left to the discretion of physician of each hospital. To date, there have been no randomised trials or nation-wide studies regarding this issue. Accordingly, there are no guidelines whether to provide antibiotics for patients after cardiac arrest due to the lack of data. Approximately, 45 % of the patients received antibiotics in the present study. Although analysis of the baseline patient characteristics in the unmatched group showed that antibiotics were used in a greater proportion of patients with more severe status after cardiac arrest (i.e. those at greater risk of death and more likely to require defibrillation upon admission, PCI, IABP, ECMO, catecholamines, antiarrhythmic medications, or blood products), one-to-one propensity score-matching successfully balanced the characteristics of the antibiotic and control groups. Although patients receiving antibiotics appeared less likely to die than did similar patients who did not receive antibiotics (mortality rates 33.0 and 29.9 %, respectively), this difference was not statistically significant.

Similar results were observed in the instrumental variable analysis, which also found that there was no significant reduction in 30-day mortality associated with the receipt of antibiotics. When hospitals have highly consistent antibiotic-use policies for post-cardiac arrest patients treated with a TTM strategy, decisions regarding antibiotic use may be made independently of individual patient characteristics, including unmeasured variables, as OHCA occurs independent of the location of the admitting hospital. In this situation, the hospital’s antibiotics use policy may act as an instrumental variable, thereby setting the conditions for a ‘natural experiment’ that allows for an unbiased estimate of risk in patients with OHCA undergoing TTM. Considering the results of the propensity score and instrumental variable analyses, early antibiotic-administration appears to have little influence on survival during TTM after OHCA. Our negative results of the present study may have resulted from a lack of statistical power. However, the absolute difference in 30-day mortality in the propensity-matched analysis was 3.1 %, thereby suggesting that at least 7272 patients (3636 patients per group) would be required to show a significant difference in future trials (α error = 0.05, 1-β [power] = 0.8).

The use of ECMO in the intensive care unit has substantially increased over the past ~10 years [17, 29, 30], including during cardiopulmonary resuscitation (CPR) and afterwards [16, 31, 32]. The results of a recent meta-analysis by Xie et al. [33] suggested that ECMO has the potential to improve outcomes after cardiac arrest; however, they also suggested that ECMO is associated with significant complication rates, including infection, which must be incorporated into the risk-benefit analysis when considering treatment [33]. The latest resuscitation guidelines state that extracorporeal CPR is a reasonable rescue therapy for selected patients with cardiac arrest when initial conventional CPR is failing; however, they provide no guidance regarding the role of antibiotic administration [6, 7].

A low cardiac output state combined with high systemic vascular resistance results in poor systemic perfusion in OHCA patients who successfully achieve ROSC. Post-cardiac arrest acute kidney injury occurs in more than 50 % of cardiac arrest patients [3]. Potential treatments to counteract this severe hemodynamic status and acute renal failure include IABP and CRRT. Although these devices themselves may increase the risk of infection, the use of prophylactic antibiotics is controversial [18]. No studies have evaluated the relationship between early antibiotic administration and mortality among OHCA patients requiring these invasive life-supporting interventions.

We found that patients with cardiogenic OHCA who required ECMO, IABP, or CRRT might benefit from early antibiotic treatment in our propensity score and instrumental variable analyses. Early antibiotic administration may have the greatest therapeutic benefits in patients with the most severe OHCA patients with TTM. Grimaldi et al. [34] recently reported that nearly half of the patients with post-cardiac arrest shock had high blood endotoxin concentration in the first 12 h following ROSC. They also reported that endotoxemia was associated with shock duration, mean daily dose of vasopressors and organ failure [34]. Thus, among those patients with OHCA and severe hemodynamic disturbance to require ECMO, IABP, and/or CRRT, early antibiotics administration may have the potential to improve outcomes.

Another explanation could be that ECMO, IABP, and/or CRRT themselves increase the risk of nosocomial infection. Patients on ECMO for extracorporeal CPR reportedly developed 24.7 infections per 1000 ECMO days according to data from the Extracorporeal Life Support Organization Registry [35], and these infections were associated with increased mortality. Nevertheless, the Task Force does not currently recommend routine antibiotic administration during ECMO, due to lack of evidence of its efficacy [17]. Furthermore, previous studies showed that the incidence of catheter-related bacteremia in patients who required hemodialysis ranged between 0.6 and 6.5 episodes per 1000 catheter days, and increased linearly with the duration of catheter use [18, 36]. Although we cannot infer a robust cause-and-effect relationship from this study, we speculate that there may be an association between early antibiotic administration and improved survival in OHCA patients requiring ECMO, IABP, and/or CRRT during TTM. We believe that our nationwide retrospective data will be useful when planning for future international trials.

Although the incidence of pneumonia after OHCA is inconsistent across previous studies, the incidence of pneumonia in the present report may be lower than that of previous reports outside Japan [8, 10, 15, 20, 21]. This may be due to several reasons. Firstly, because of its retrospective design, the diagnosis of pneumonia was assigned to the doctors in charge of each 371 hospitals. Thus, there might be some under-reporting (or over-reporting) in the current results. Secondly, in the present study, we included only cardiogenic OHCA patients that underwent TTM. In Japan, during the study period, most TTMs were managed with a milder target temperature for a longer period (e.g. duration of cooling for >2 days in 34 °C and >2 days for rewarming [32, 37]), than the recommendations in international guidelines published during the study period (i.e. cooling to 32 °C to 34 °C for 12 or 24 h [38]). Due to these differences, both the incidence and recognition of pneumonia by the attending doctors may have decreased. Further studies are required to clarify our speculations.

Our study has several limitations. Firstly, although we evaluated nationwide data using a propensity score and instrumental variable analyses, the study was conducted retrospectively. Therefore, a cause-and-effect relationship cannot be established. Large randomized trials are necessary to confirm our findings; however, such studies are challenging in this vulnerable population. Indeed, the mortality rate exceeds 50 % in those who underwent ECMO. Secondly, a limited number of covariates could be analysed as the database did not include several important factors, particularly pre-hospital data including whether the OHCA was witnessed by a bystander, the quality of resuscitation given, whether resuscitation was dispatcher-assisted, and how the emergency services treated the patients [39]. Although we endeavoured to compensate for these potential unmeasured confounders using instrumental variable analyses, further studies are required. Thirdly, we evaluated cardiogenic OHCA patients who underwent therapeutic hypothermia without pneumonia on hospital arrival in the present study. However, since pneumonia often presents gradually after admission, there might be some undiagnosed or underreported pneumonia. In addition, we could not identify catheter-related infections from the current database. Fourthly, the DPC is an administrative database with information input in relation to patient discharge. We could not follow up patients after discharge from the hospital because this information was not available in the database. Finally, we evaluated only adults with cardiogenic OHCA treated by TTM. Our results cannot be generalized to the use of prophylactic or early administration of antibiotics for children, adults with non-cardiogenic OHCA (especially who already had pneumonia on admission), in-hospital cardiac arrest patients, or those not treated with TTM.

## Conclusions

Although there was no significant association between the use of early antibiotics administration and mortality in a large cohort of patients with cardiogenic OHCA undergoing TTM, antibiotics appear to improve survival in a subgroup of patients that require ECMO. Further studies are required to confirm our results.

## Abbreviations

CPR: Cardiopulmonary resuscitation
CRRT: Continuous renal replacement therapy
ECMO: Extracorporeal membrane oxygenation
IABP: Intra-aortic balloon pumping
OHCA: Out-of-hospital cardiac arrest
PCI: Percutaneous coronary angiography/intervention
TTM: Targeted temperature management
vf: ventricular fibrillation
